# Editorial: Plant secondary metabolites: Potential therapeutic implications in neuropsychiatric disorders

**DOI:** 10.3389/fnbeh.2023.1153296

**Published:** 2023-03-03

**Authors:** Juan Francisco Rodríguez-Landa, Damiana Scuteri, Lucía Martínez-Mota

**Affiliations:** ^1^Laboratorio de Neurofarmacología, Instituto de Neuroetología, Universidad Veracruzana, Xalapa, Mexico; ^2^Facultad de Química Farmacéutica Biológica, Universidad Veracruzana, Xalapa, Mexico; ^3^Pharmacotechnology Documentation and Transfer Unit, Section of Preclinical and Translational Pharmacology, Department of Pharmacy, Health and Nutritional Sciences, University of Calabria, Rende, Italy; ^4^Regional Center for Serious Brain Injuries, S. Anna Institute, Crotone, Italy; ^5^Dirección de Investigaciones en Neurociencias, Instituto Nacional de Psiquiatría Ramón de la Fuente Muñiz, Ciudad de México, Mexico

**Keywords:** aromatherapy, antidepressant, anxiolytic, medicinal plant, behavioral pharmacology

According to reports by the World Health Organization, neuropsychiatric disorders, particularly anxiety and depression, are the leading cause of disability around the world (Charlson et al., 2019). Treatment strategies-based on synthetic anxiolytic drugs include the use of benzodiazepines and antidepressant drugs endowed with anxiolytic-like activity as fluoxetine, while the treatment of depression disorders includes the use of selective reuptake inhibitors of serotonin, norepinephrine, norepinephrine/dopamine, and tricyclic antidepressants, among others (Garakani et al., 2020; Stachowicz and Sowa-Kućma, 2022). Although those anxiolytic and antidepressant drugs are effective in the management of some neuropsychiatric disorders, they have important limitations as several side effects, slow response onset, and low efficacy in particular cases as post-partum and menopause, among others (Graziottin and Serafini, 2009; Wang et al., 2018; Brown et al., 2021; Saha et al., 2021). In this sense, some patients ask for complementary or alternative therapies to improve their mental health, among which, medicinal plants are some of the most used resources (Kris-Etherton et al., 2021; Wang et al., 2023).

Ancient cultures around the world describe therapeutic effects of medicinal plants to relieve physical pain, inflammation, and heal mental states associated with culture-bound syndromes. It is estimated that ~80% of the global population consumes herbal medicinal products as a primary source of healthcare (Hamilton, 2004; Chen et al., 2016), which in most cases lack controlled evaluations that validate their therapeutic use (Sarris et al., 2021). Thus, there is a real need to scientifically characterize the pharmacological, toxicological, and potential therapeutic effects of vegetal species and secondary metabolites through multidisciplinary controlled assays. Modern technological tools, molecular and cellular protocols, and validated animal models may be useful for the identification of unknown metabolites with psychoactive activity for the treatment of neuropsychiatric disorders, and pain control, among other afflictions.

Recent studies from preclinical and clinical research have identified pharmacological properties of some molecules isolated from extracts of medicinal plants that target neurotransmitters pathways such as serotonergic, dopaminergic, GABAergic, and noradrenergic systems (Rodríguez-Landa et al., 2022). These molecules promote the activation of neurotrophic factors such as the brain derived neurotrophic factor (BDNF) and nerve growth factor (NGF) improving neuronal plasticity and brain connectivity (German-Ponciano et al., 2022). Particularly, polyphenols reduce oxidative stress and neuroinflammation, that play a role in the impairment of the appropriate functioning of cells and tissues (Jia et al., 2022). Those effects are considered part of the mechanism of action underlying the anxiolytic, antidepressant, and beneficial effects of polyphenols also on other neuropsychiatric disorders (Morris et al., 2021).

In this Research Topic, interesting studies are included, from potential tools to study the anxiolytic- and antidepressant-like effects of molecules contained in plants, to the effects of some plant secondary metabolites, as flavonoids, when they are microinjected in specific brain structures involved in neuropsychiatric disorders. Cueto-Escobedo et al. contributed with a review of the effects of natural products as anxiolytics using zebrafish as a model of anxiety. This proposal arises from an ethical responsibility, for example, search for new alternatives to reduces the number of animals in research, and from the ability of zebrafish to exhibit defensive behaviors sensitive to anxiolytic drugs. The authors compare behavioral, neurochemical, and molecular similarities between models, emphasizing the advantages of the zebrafish use in reducing costs and time in evaluating the potential therapeutic effects of extracts and molecules from medicinal plants.

Changes in the endocrine milieu and stress are potential etiological factors for neuropsychiatric disorders. In this regard, Cervantes-Anaya et al. verified the antidepressant-like effect of the aqueous extract of pomegranate (*Punica granatum*) and evaluated the effect of polyphenols found in the extract, punicalagin and ellagic acid, in ovariectomized rats. The authors concluded that the extract of pomegranate and its active compounds exert antidepressant-like effects, probably associated with antioxidant activity as they attenuated oxidative damage and prevented cellular dysfunction in ovariectomized rats. These results could impact the development of natural strategies to ameliorate depressive symptoms in menopausal women as it has been previously suggested (Valdés-Sustaita et al., 2017). Rodríguez-Landa et al., evaluated the effect of flavonoid chrysin microinjected into the dorsal hippocampus, a brain structure that participates in the regulation of anxiety, on anxiety-like behavior considering the ovarian cycle phases. Interestingly, they found that the anxiolytic-like effect of chrysin is dependent on the hormonal status during the ovarian cycle and discuss the critical role of GABA_A_ receptor in the dorsal hippocampus to modulate anxiety-like responses associated with a context. These findings could contribute to the development of natural therapies destined to the management of anxiety symptoms associated with premenstrual dysphoric syndrome in women as it has been suggested in previous studies (Rodríguez-Landa et al., 2021).

On the other hand, Contrada et al. present the beneficial effects of aromatherapy in stroke patients. In this frame the essential oil of bergamot (BEO) is highlighted since it provided strong evidence of anti-nociceptive, anti-neuropathic, and anxiolytic-like flumazenil-insensitive properties in reliable models and rigorous research (Scuteri et al., 2021). The gathered data form the rational basis for the clinical study (Scuteri et al., 2022) of its engineered form on agitation and pain in dementia patients often neglected from trials (Scuteri et al., 2020). Finally, Zepeda et al. present an opinion article about the use of St. John's wort (*Hypericum perforatum*) in the treatment of perinatal depression. They report some beneficial effects on depression symptoms, but also highlight the potential side effects to the mother and the fetus as it was reported in the general population from a few years ago (Rodríguez-Landa and Contreras, 2003). The effects of specific secondary metabolites, the individual variations in women, the consumption of different commercial presentations of the herbal product, and the lack of clinically controlled studies stand out among factors that are critically discussed in this proposal.

Collectively, investigating the pharmacological effects of plants, their derivatives, and their isolated compounds through preclinical and clinical research, is a critical step to obtain deep insight into the effectiveness and safety of vegetal species (and their components) with potential therapeutic effects in particular groups of patients with neuropsychiatric disorders. We are confident that this Research Topic will arise interest in the scientific community and contribute to biomedical and pharmaceutical research for the development of novel drugs for the treatment of neuropsychiatric disorders.

## Author contributions

All authors listed have made a substantial, direct, and intellectual contribution to the work and approved it for publication.
